# Lipid profile and left ventricular geometry pattern in obese children

**DOI:** 10.1186/s12944-020-01285-9

**Published:** 2020-05-26

**Authors:** Bojko Bjelakovic, Claudia Stefanutti, Vladimir Vukovic, Nebojsa Kavaric, Ljiljana Saranac, Aleksandra Klisic, Stevo Lukic, Sanja Stankovic, Maja Jovic, Sergej Prijic, Marko Bjelakovic, Maciej Banach

**Affiliations:** 1 Clinical Center, Nis, Zorana Djindjica 48 Boulevard, Nis, 18000 Serbia; 2 Medical faculty, University of Nis, Nis, Serbia; 3 Extracorporeal Therapeutic Techniques Unit, Lipid Clinic and Atherosclerosis Prevention Centre, Immunohematology and Transfusion Medicine, Department of Molecular Medicine, “Sapienza” University of Rome, “Umberto I” Hospital, Rome, Italy; 4 Institute for Biomedicine, Eurac Research, Affiliated Institute of the University of Lübeck, Bolzano, Italy; 5 Primary Health Care Center, Podgorica, Montenegro; 6 Clinic of Neurology-Clinical Center, Nis, Serbia; 7 Mother and Child Health Institute, “Dr Vukan Cupic,” Belgrade, Nis, Serbia; 8 Center for Medical Biochemistry, Clinical Center of Serbia, Belgrade, Serbia; 9 Medical faculty, University of Belgrade, Belgrade, Serbia; 10 Mother and Child Health Institute of Serbia, Belgrade, Serbia; 11 Clinic of Pulmology, Clinical Center, Nis, Serbia; 12 Department of Hypertension, Chair of Nephrology and Hypertension, Medical University of Lodz, 281/289 Rzgowska St., 93-338 Lodz, Poland; 13 Polish Mother’s Memorial Hospital Research Institute (PMMHRI), Lodz, Poland; 14 Cardiovascular Research Centre, University of Zielona Gora, Zielona Gora, Poland

**Keywords:** Triglycerides, Obesity, Children, Left ventricular mass index, Relative wall thickness

## Abstract

**Background:**

Left ventricular hypertrophy (LVH) is an important risk factor for cardiovascular and all-cause mortality. Previous studies reported conflicting results concerning the relationship between serum lipid levels and left ventricular geometry pattern. We sought to explore the relationship between standard serum lipid profile measures with left ventricular geometry pattern in obese children.

**Patients and methods:**

In this cross-sectional study, a total of 70 obese children were examined. Fasting blood samples were taken to measure total cholesterol, low density lipoprotein cholesterol (LDL-C), high density lipoprotein cholesterol (HDL-C), triglycerides (TGs), glucose, and insulin. Based on these values *TG/HDL* ratio, BMI and HOMA index were calculated. We also measured the average 24-h ambulatory systolic blood pressure (SBP) and two-dimensional (2/D) transthoracic echocardiography was performed to determine left ventricular mass index (LVMI) and relative wall thickness (RWT). Multiple regression analyses were conducted to explore relationships between study variables and the LVMI or RWT as outcome variables. The final model with LVMI included TG/HDL ratio, BMI, 24 h-average SBP, age and sex, while for the RWT we included BMI, insulin, age and sex.

**Results:**

Our study included 70 children (65.71% boys and 34.29% girls) median age (14 years, IQR = 12–16)." We demonstrated independent and positive association of TG/HDL ratio, BMI and 24 h-average SBP with LVMI (effect = 3.65, SE = 1.32, *p* < 0.01; effect = 34.90, SE = 6.84, p < 0.01; effect = 0.32, SE = 0.12, p < 0.01, respectively). On the other hand, in model with RWT as outcome variable, only BMI and insulin were significantly linked (BMI: effect = 13.07, SE = 5.02, *p* = 0.01 Insulin: effect = 2.80, SE = 0.97).

**Conclusion:**

Increased TG/HDL ratio in obese children is associated with the development of eccentric left ventricular hypertrophy while increased BMI and insulin were associated with concentric left ventricular hypertophy.

## Introduction

It is well established that the left ventricular hypertrophy (LVH) is an important risk factor for cardiovascular and all-cause mortality and morbidity [1–3]. A few cross-sectional studies have shown that male sex, age, genetics, hemodynamic overload, obesity, hormonal and metabolic factors correlate with LVH [4, 5]. Previous studies have reported conflicting results concerning the relationship between serum lipid levels and left ventricular geometry pattern, both in general population and patients with cardiovascular risk [6–9]. There are several *proposed* mechanisms through which hyperlipidemia may contribute to the left ventricular remodeling and hypertrophy These are a) accumulation of lipids in or around myocytes b) l*ocal and systemic inflammation c)* insulin resistance d) oxidative stress, especially mitochondrial oxidative stress e) neuro-humoral effects including both autonomic-nervous-system dysfunction and other hormonal influences, mainly the renin-angiotensin-aldosterone system (RAAS) [10–15].

Left ventricular hypertrophy is a common echocardiographic abnormality in obese children which can’t be explained solely by hemodynamic mechanisms. On the other hand, altered lipid profile including elevated triglycerides (TG), VLDL (Very-Low-Density Lipoprotein) total cholesterol (TC) and low High-Density Lipoprotein (HDL) cholesterol is a frequent metabolic abnormality possible contributing to the development of cardiac hypertrophy in obese children [16, 17].

Recent studies suggest that besides direct accumulation of lipids in or around myocytes, other signaling pathways elicited by atherogenic dyslipidemia play a significant role in developing cardiac hypertrophy. It was shown that VLDL can promote aldosterone overproduction which in turn may induce LV remodeling independently of its hemodynamic effects on systemic BP [14, 18] In addition, the results of several cross-sectional and longitudinal studies reported that increased plasma aldosterone is associated with decreased HDL-C and increased triglycerides in patients with metabolic syndrome [19].

Related to mentioned researches this study aimed to explore the potential relationship between traditional cardiovascular risk factors and serum lipoprotein profile, with the left ventricular geometry pattern in obese children.

## Material and methods

### Study design

We conducted a cross-sectional survey on an initial sample of 70 obese children consecutively referred by the local and regional health units to our institution (Clinic of Pediatrics, Clinical Center Nis, Serbia) for further clinical evaluation of the obesity-related cardiovascular complication, from January 2016 to June 2018. The study inclusion criteria were that none of the children had positive history of antihypertensive drug treatment or other chronic diseases.

The study was approved by the Ethical Committee for Medical Research, (protocol number 01–9002-6). W*e obtained informed consent* from the parents of children included in the study. Obesity was defined as a BMI at or above the 95th percentile for children and teens of the same age and sex according to the reference values published by the Centers for Disease Control and Prevention [20]. Body mass index (BMI) was calculated as weight (in kg) divided by height (in m) squared.

### Covariates

In all study participants we measured: a) Standard lipid profile (Cholesterol, Triglycerides, LDL-C, and HDL-C) using UniCel DxC Systems (Beckman Coulter, Inc); b) Insulin level - using a chemiluminescent assay (AccessDxI800; Beckman Coulter Inc., Brea, CA); c) plasma glucose level. All blood specimens were taken after an overnight fasting. The ambulatory 24 h SBP was measured using the Scanlight measurement device (Scanlight III; I.E.M GmbH, Germany), which, according to certificate of equivalence DIN EN 9001:2000, 13,485, CMDCAS, is functionally and technically identical to the Mobil-O-Graph New Generation device that was validated in children.

The HOMA index was calculated as the product of the fasting plasma insulin level (microU/mL) and the fasting plasma glucose level (mmol/L) divided by 22.5. We further calculated the TG/HDL ratio in every participant.

### Left ventricular structure

We studied left ventricular structure (geometry) through 2-D standard echocardiography and the left ventricular myocardial mass index (LVMI) was measured by M-mode echocardiography using the Devereux formula indexed by body height to the power of 2.7.

Relative wall thickness (RWT) was defined as LV wall thickness + septal thickness relative to the internal dimensions of the LV [21].

LV geometric patterns were defined based on whether RWT and/or LVM are normal versus increased: normal geometry (LVM and RWT are normal), concentric remodeling (increased RWT but normal LVM), concentric hypertrophy (LVM and RWT are increased) and eccentric hypertrophy (increased LVM with normal RWT). All echocardiographic measurements were performed in triplicate by the same specialized cardiologist, who was unaware of the subject’s BP, using the Acuson Sequoia 256 Cardiac Ultrasound Machine, 2,5–3,5 MHz probe.

### Statistical analysis

We performed descriptive analyses of the demographic and clinical characteristics of the participants using mean, median, range and standard deviation for continuous and frequencies for the categorical variables. The assumption of normality was checked through the Shapiro-Wilk test and variables with a skewed distribution (age, BMI, insulin, TG, HDL-C, TG/HDL and HOMA) were logarithmically transformed to achieve normal distribution. The strength of correlation between different variables was evaluated using the Pearson’s linear correlation. Multiple regression analyses with stepwise selection were conducted to study relationships between age, sex, BMI, average 24 h - SBP, serum lipoprotein concentration and LVMI or RWT as outcome variables. Only variables attaining statistical significance in the univariate analysis at a 0.05 level as well as variables known from previous research to be associated with LVMI and RWT were kept in the final model. The *p*-value < 0.05 was considered statistically significant. All statistical analyses were performed using SPSS version 20 (SPSS, Chicago, IL, USA).

## Results

### Characteristics

Our study included 70 children (65.71% boys and 34.29% girls) with median age (14 years, IQR = 12–16) that were referred to our institution for further clinical assessment. Median BMI was 28.65 kg/m^2^ (IQR = 26.7–31.3) while 24 h average SBP was 123.77 mmHg (SD = 8.78). Median cholesterol was 4.4 mmol/L (IQR = 3.96–4.74), median triglycerides 1.19 mmol/L (IQR = 0.78–1.86) while LDL-C and HDL-C had median values of 2.4 mmol/L (IQR = 1.9–2.7) and 1.3 mmol/L (IQR = 1.08–1.41), respectively. From these values we calculeted the TG/HDL ratio which had median of 0.86 (IQR = 0.58–1.57). Insulin measurement was available for 58 (82.86%) children in which a median value was 25 μU/mL (IQR = 18–47). Left ventricular mass index was 46 g/m^2.7^ (IQR = 42–55) while the relative wall thickness was 36.5% (IQR = 31–41). Eccentric left ventricular hypertrophy (LVH) was found in 14 (20%) participants, concentric LVH in 10 (14.29%) and concentric remodeling in 10 (14.29%), while 36 (51.43%) had normal left ventricular geometry. The demographic and clinical characteristics of all participants are described in Table 1.

**Table 1:** Demographic and clinical characteristics of the study participants

	**Mean**	**SD**	**Median**	**IQR**	**Min**	**Max**
Age, years	13.77	2.32	14	12–16	9	17
Sex, n (%)	70 (100)					
Male, n (%)	46 (65.71)					
Female, n (%)	24 (34.29)					
BMI, kg/m^2^	29.38	4.26	28.65	26.7–31.3	20.1	40.4
BMI z-score	2.24	0.56	2.27	1.93–2.57	1.05	3.52
24 h-average SBP, mm Hg	123.77	8.78	124	119–130	101	145
**Laboratory assessment**						
Cholesterol, mmol/L	4.36	0.79	4.4	3.96–4.74	2.85	6.91
Triglycerides, mmol/L	1.42	1.01	1.19	0.78–1.86	0.2	7
LDL, mmol/L	2.38	0.64	2.4	1.9–2.7	0.99	4.2
HDL, mmol/L	1.30	0.30	1.3	1.08–1.41	0.78	2.12
Glucose, mmol/L	5.23	0.59	5.2	4.9–5.6	3.8	6.8
Insulin, μU/mL	39.15	34.14	25	18–47	3	163
HOMA index	9.12	8.95	5.72	4.11–11.2	0.51	41.51
TG/HDL ratio	1.17	0.84	0.86	0.58–1.57	0.16	4.57
**Echocardiographic assessment**						
LVMI, g/m^2.7^	47.85	9.97	46	42–55	25	75
RWT, %	36.40	6.36	36.5	31–41	23	51
**Left ventricular geometry classification,** n (%)	70 (100)					
Normal geometry, n (%)	36 (51.43)					
Eccentric LVH, n (%)	14 (20)					
Concentric LVH, n (%)	10 (14.29)					
Concentric remodeling, n (%)	10 (14.29)					

Abbreviations: *SD* standard deviation, *IQR* inter-quartile range, *BMI* body mass index, *LDL* Low Density Lipoprotein, *HDL* High Density Lipoprotein, *TG* Triglycerides, *HOMA* Homeostatic model assessment index, *RWT* relative wall thickness, *LVMI* Left ventricular mass index, *BP* blood pressure

### Correlations

Based on the analysis on entire sample of children, triglycerides positively correlated with left ventricular mass index (r = 0.32, *p* < 0.01 while TG/HDL ratio positively correlated with left ventricular mass index and relative wall thickness (r = 0.33, *p* < 0.01; r = 0.24, *p* < 0.05, respectively). The other serum lipoprotein levels did not correlate with left ventricular mass index and left ventricular relative wall thickness.

Of other examined variables we found positive correlation of BMI and LVMI and RWT (r = 0.55 *p* < 0.01; r = 0.39, *p* < 0.01, respectively); 24 h ambulatory average SBP and LVMI (r = 0.35, *p* < 0.01) and RWT (r = 0.33, *p* < 0.01) while insulin and HOMA index positively correlated only with the RWT (r = 0.41, *p* < 0.01; r = 0.43, *p* < 0.01, respectively) (Table 2).

**Table 2:** Correlation between serum lipoprotein fractions, BMI, insulin, 24-h average SBP, HOMA with LVMI and RWT

	**LVMI**		**RWT**	
	**correlation**	***p*-value**	**correlation**	***p*-value**
Cholesterol, mmol/L^1^	−0.19	0.23	−0.08	0.52
Triglycerides, mmol/L^1^	0.32	< 0.01**	0.19	0.13
LDL, mmol/L	−0.003	0.98	0.04	0.75
HDL, mmol/L^1^	−0.16	0.21	−0.23	0.06
TG/HDL ratio [1]	0.33	< 0.01**	0.24	< 0.05*
BMI [1]	0.55	< 0.01***	0.39	< 0.01**
Insulin, μU/mL^1^	0.11	0.4	0.41	< 0.01**
24 h-average SBP	0.35	< 0.01**	0.33	< 0.01**
HOMA index [1]	0.13	0.36	0.43	< 0.01**

^1^log transformed

Significance levels are given for *p < 0.05; **p < 0.01; ***p < 0.001

Abbreviations: *BMI* body mass index, *LDL*Low Density Lipoprotein, *HDL* High Density Lipoprotein, *TG* Triglycerides, *LVMI* Left ventricular mass index, *SBP* systolic blood pressure, *RWT* relative wall thickness

### Regression analyses

Results from the linear regression analyses on the relationship between LVMI and RWT as an outcome variables and serum lipoproteins, BMI, Insulin, 24 h-average SBP and HOMA index are presented in Table 2.

Using multiple regression analyses with stepwise adjustment in a model with LVMI as a dependent variable and age, gender, BMI, 24 h-average SBP, TC, LDL-C, HDL-C, TG/HDL ratio and insulin as independent parameters, the TG/HDL ratio showed statistically significant independent positive association with LVMI (effect = 3.65, SE = 1.32, *p* < 0.01). Besides, BMI and the 24 h-average SBP also showed independent positive association with LVMI (BMI effect = 34.90, SE = 6.84, p < 0.01; 24 h-average SBP: effect = 0.32, SE = 0.12, p < 0.01), Table 3.

**Table 3:** Multivariable regression results of LVMI and RWT in total sample of children

**LVMI**				
**Variable**	**Effect**	**SE**	**95% CI**	***p*-value**
Age [1]	−16.20	5.62	−27.43 to −4.95	< 0.01**
Sex	5.69	1.89	1.93 to 9.47	< 0.01**
TG/HDL ratio [1]	3.65	1.32	1.01 to 6.29	< 0.01**
BMI [1]	34.90	6.84	21.21 to 48.58	< 0.01***
24 h-average SBP	0.32	0.12	0.09 to 0.55	< 0.01**
Constant	−71.09	22.38	−115.84 to −26.34	< 0.01**
**RWT**				
**Variable**	**Effect**	**SE**	**95% CI**	***p-value***
Age [1]	2.44	4.16	−5.90 to 10.78	0.56
Sex	0.68	1.50	−2.32 to 3.68	0.65
BMI [1]	13.07	5.02	3.00 to 23.13	0.01*
Insulin [1]	2.80	0.97	0.85 to 4.74	< 0.01**
Constant	−23.68	16.54	−56.87 to 9.50	0.16

^1^log transformed

Significance levels are given for *p < 0.05; **p < 0.01; ***p < 0.001

Abbreviations: *SE* standard error, *95% CI* 95% confidence interval, *BMI* body mass index, *LDL* Low Density Lipoprotein, *HDL* High Density Lipoprotein, *TG* Triglycerides, *RWT* relative wall thickness, *LVMI* Left ventricular mass index, *SBP* systolic blood pressure

Since the TG also demonstrated positive correlation with the LVMI, we tried another analysis using TG instead of the TG/HDL ratio in the regression model and did not obtain stitisticaly significant result (results not shown).

On the other hand, in a model with RWT as a dependent variable, and age, gender, BMI, 24 h- average SBP, cholesterol, HDL-C, LDL-C, TG/HDL and insulin as independent parameters, only BMI and insulin showed statistically significant independent positive associations with RWT (BMI: effect = 13.07, SE = 5.02, *p* = 0.01; insulin: effect = 2.8, SE = 0.97, *p* < 0.01), as reported in Table 3.

## Discussion

Based on the obtained results we showed that in obese children TG/HDL ratio was postively associated with eccentric left ventricular hypertrophy, and BMI and insulin levels are postively associated with concentric left ventricle hypertrophy.

To data only a few studies have been published focusing on the relationship between serum lipid and lipoprotein levels and left ventricular mass and geometry both in children and adults.

Sundstrom et al. showed that dyslipidaemia and unfavorable fatty acid profile at age 50 predicted the prevalence of LVH at age 70, to a similar degree as hypertension and obesity [6]. One autoptic study of human hearts indicated that the fat deposition in the left ventricle constitutes a direct risk of cardiac hypertrophy [22]. In addition, by using proton magnetic resonance spectroscopy Kankaanpää et al. demonstrated accumulation of TG in myocardium of moderately obese subjects. They reported that free fatty acid levels were a significant predictor of LV mass, whereas myocardial and epicardial fat were more strongly related to LV work and mechanical load [23]. Likewise, Kaminaga and others concluded that hypertrophic cardiomyopathy may be associated with the presence of myocardial fat in the LV with a thickened wall [24]. One preclinical study has recently shown that increased level of glycosphingolipids, may be an independent factor contributing to cardiac hypertrophy [8].

As for children, Dabas et al. reported no correlation between lipids and LV parameters in adolescents with type 1 diabetes [25]. On the other hand Di Bonito at al. identified a positive association between TG/HDL ratio and left ventricular mass and RWT independently of other cardiovascular (CV) risk factors in obese children which is partly in line with our results [26].

In this respect, our findings are also in agreement with those reported by Al-Daydamony and El-Tahlawi et al., who found a significant positive correlation between LVMI and TG levels in patients with metabolic syndrome without hypertension [27].

The disappearance of any relationship between TG/HDL ratio with RWT in multivariable regression equation correlation, indicate that BMI and 24 h-average SBP rather than insulin resistance (of which TG/HDL ratio is a surrogate marker) are associated with concentric left ventricular hypertrophy [9, 28–30].

Although the pathophysiological explanation of these results is not straightforward, and we didn’t measure aldosterone as well as VLDL levels in our patients, we can’t exclude the effects of potential signaling pathways activated by the VLDL which resulted in aldosterone overproduction and development of left ventricular hypertrophy. It is now appreciated that left ventricular hypertrophy (LVH) in hypertensive obese patients is not only mediated via clear-cut increase in blood pressure but also by various neuro-hormonal and metabolic factors that independently exert trophic effects on myocytes and non-myocytes in the heart [31, 32]. Experimental and clinical evidence clearly indicates that protracted exposure to inappropriately elevated aldosterone levels induces cardiac inflammation and interstitial fibrosis leading to a maladaptive remodeling in the heart and changes in left ventricular structure and function, regardless of the level of blood pressure. In particular, recent studies reported a possible involvement of aldosterone as a trigger of the activation of p38 mitogen-activated protein kinase (MAPK) and nicotinamide riboside kinase (nRK1/2) which have been involved in signal transduction pathways associated with cardiac hypertrophy [33, 34].

Recently it was shown that adipose tissue, via secretion of factors such as leptin, stimulates adrenal aldosterone secretion as well as that VLDL might induce aldosterone overproduction via induction of steroidogenic acute regulatory (StAR) protein and aldosterone synthase (CYP11B2) expressio n[35, 36]. Given the fact that VLDLs are major transporters of TG accounting for approximately 85% of their weight, as well as the strong positive correlation between TG levels and TG/HDL ratio (r = 0,885) in our study, we can’t exclude the possible link between atherogenic dyslipidemia (high TG/HDL ratio), TG, aldosterone and LVH or TG -VLDL - aldosterone - LVH continuity in obese children.

Furthermore, Goodfriend et al. described a negative association between aldosterone concentration and HDL-C and a positive correlation between aldosterone and triglycerides in a group of 30 volunteers. Inverse association between aldosterone levels with HDL-C in the general population was also confirme by Hannich et al. [19, 36] They speculated that HDL-C may inhibit adrenal aldosterone secretion by modulating the sensitivity of the adrenal zona glomerulosa [36].These results may possibly explain our finding that only TG/HDL ratio but not only TG levels were associated with eccentric LVH.

We would also like to point out the findings of Brady et al. who recently reported for the first time that serum aldosterone activity was higher in obese children with LVH when compared to those without LVH. Likewise, Somloova et al. found in patients with primary aldosteronism that HDL-C was markedly lower and BMI and TG were significantly higher in those having idiopathic hyperaldosteronism (IHA) than in patients with aldosterone-producing adenoma (APA). In addition, the metabolic syndrome was more prevalent in IHA than in APA [37].

In our opinion high TG/HDL ratio (indirectly high VLDL levels and low HLD levels) which has been shown to be associated with increased aldosterone concentration may be one possible explanation of these results. Since the magnitude of aldosterone elevation in obese children has not been object of our scientific research, we guess that further research, including direct measurement of serum aldosteron, VLDL levels and HDL-C levels may identify new subset of at risk obese patients for LVH, as well as novel targets for the treatment of obese children with LVH but without hypertension.

An alternative *explanation of our results might be explained by facts that high* TG levels (indirectly high VLDL levels) may affect the synthesis of pro-inflammatory cytokines TNF-α further promoting adverse cardiac remodeling, characterised by increased total are metalloproteases (MMP) activity and increased fibrosis [38]. The functional crosstalk between angiotensin II (Ang II) and tumor necrosis factor (TNF)-α has also been shown to cause adverse left ventricular remodeling and hypertrophy in hypertension [9, 39].

Our second finding that BMI and insulin levels are associated with developing concentric left ventricle geometry pattern in obese children was recently confirmed using magnetic resonance imaging in the multi-ethnic study of atherosclerosis and is accordance with results of Gubbio study in adults and results of Urbina et al. study in children [40].

### Strengths and limitation

There are several limitations of this study including small number of participants as well as the, lack of control group of metabolically healthy obese children. Also, we cannot exclude the effects of some potential genetic determinants and environmental effects on left ventricular geometry that were not considered in our study which may have ultimately influenced the correlations reported here. However, this is only the first report suggesting a possible existence of TG -VLDL - aldosterone - LVH continuity in obese children, and a possible pathophysiological mechanism to explain very common practical clinical issue in pediatric cardiology such as “frequent finding of LVH in normotensive obese children”. F*urther studies with larger sample* size are required to explore the nature of the changes in cardiac structure (geometry) as a result of atherogenic dyslipidemia and possible aldosterone overproduction.

## Conclusions

To the best of our knowledge this is the first study to suggest the association of dyslipidemia asexpressed by the TG/HDL ratio with the development of eccentric left ventricular hypertrophy in obese children. The mechanisms, through which obesity causes left ventricular hypertorphy are still an area of research and require further investigations.

## Data Availability

The datasets used and analysed during the current study are available from the corresponding author on reasonable request.

## Abbreviations

TC: Total cholesterol
LDL: Low Density Lipoprotein,
HDL: High Density Lipoprotein
TG: Triglycerides
HOMA: Homeostatic model assessment index
SBP: Systolic Blood Pressure
BMI: Body mass index
LVMI: Left ventricular mass
RWT: Relative wall thickness
LVH: Left ventricular hypertrophy
VLDL: Very-Low-Density Lipoprotein
